# A recent update about seroprevalence of ovine neosporosis in Northern Egypt and its associated risk factors

**DOI:** 10.1038/s41598-021-93596-9

**Published:** 2021-07-07

**Authors:** Abdelfattah Selim, Hanem Khater, Hamdan I. Almohammed

**Affiliations:** 1 Department of Animal Medicine (Infectious Diseases), Faculty of Veterinary Medicine, Benha University, Toukh, 13736 Egypt; 2 Parasitology Department, Faculty of Veterinary Medicine, Benha University, Toukh, 13736 Egypt; 3 Department of Microbiology and Parasitology, Almaarefa University, Riyadh, 11597 Saudi Arabia

**Keywords:** Risk factors, Parasitology

## Abstract

*Neospora caninum* (Family: Sarcocystidae) is an obligate intracellular protozoan. It is one of the most critical abortifacients in ruminants. The seroprevalence of antibodies against *N. caninum* and its risk factors was investigated among 430 sheep from four North Egyptian governorates, Alexandria, Gharbia, Menofia, and Qalyubia, during the period from 2017 to 2018. Generally, the overall prevalence rate of *N. caninum* among sheep was 8.6%. The logistic regression analysis for the obtained data revealed that *N. caninum* increased significantly with age (OR = 2.4, 95% CI: 8.4–18.7) of the ewe (OR = 3.3, 95% CI: 7.6–14.9), particularly among sheep in contact with dogs (OR = 4.9, 95% CI: 7.5–14.3). Besides, locality, season, and pregnancy status of examined sheep had no significant effect on the appearance of *N. caninum* infection. the present findings confirm the presence of *N. caninum* among sheep in Egypt which probably play a role in reproductive failure in sheep. Therefore, sanitary measures and monitoring of the infection should be implemented to reduce the spreading of the infection.

## Introduction

Neosporosis is a disease caused by *Neospora caninum*, an obligate intracellular protozoan parasite^1^. It is a worldwide disease^2,3^ in which canids are the definitive hosts that shed oocysts to the environment^4^.

The domestic ruminants like sheep, goats, and cattle act as primary intermediated hosts, getting infected either horizontally through contaminated food and water with sporulated oocysts or vertically from infected mother to offspring^5^. Neosporosis have a tremendous economic impact as it causes reproductive failure in sheep^6^. Moreover, it leads to encephalitis in dogs associated with muscle atrophy and difficulty in swallowing. The serosurvey studies on the disease in ruminants including sheep are still minimal^7,8^.

In the Mediterranean area, sheep farming is favored recently for its increasingly higher production levels, which is regularly not supported by proper feeding and management, unavoidably creating stressful situations and lower resistance to opportunistic pathogens like *N. caninum*^9^. Even though the seroprevalence of *N. caninum* has been reported among sheep and goats worldwide^5,10,11^, the epidemiological situation of ovine neosporosis in Egypt is rare. There are only two studies had been reported there^12,13^ and the current situation is still unclear.

Because of the economic importance of neosporosis in ruminants and lack of treatment and vaccination, proper prevention and control strategies are the most approaches for reducing *N. caninum* infection^14,15^. Accordingly, the evaluation of related risk factors affecting its prevalence is crucial to reduce the dissemination of the disease^2,16–18^. Therefore, this study aimed to update the situation of ovine neosporosis among sheep in north Egypt and its associated risk factors after 12 years of missing information.

## Materials and methods

### Ethics statement

All procedures involving the handling and collection of samples from sheep used in this study were approved by the ethical committee for Animal Experiment of Benha University. The methods were performed in accordance with guidelines and regulations of ethical committee of faculty of veterinary medicine, Benha University and informed consent was obtained from owners.

### Study area description

**Figure 1:**
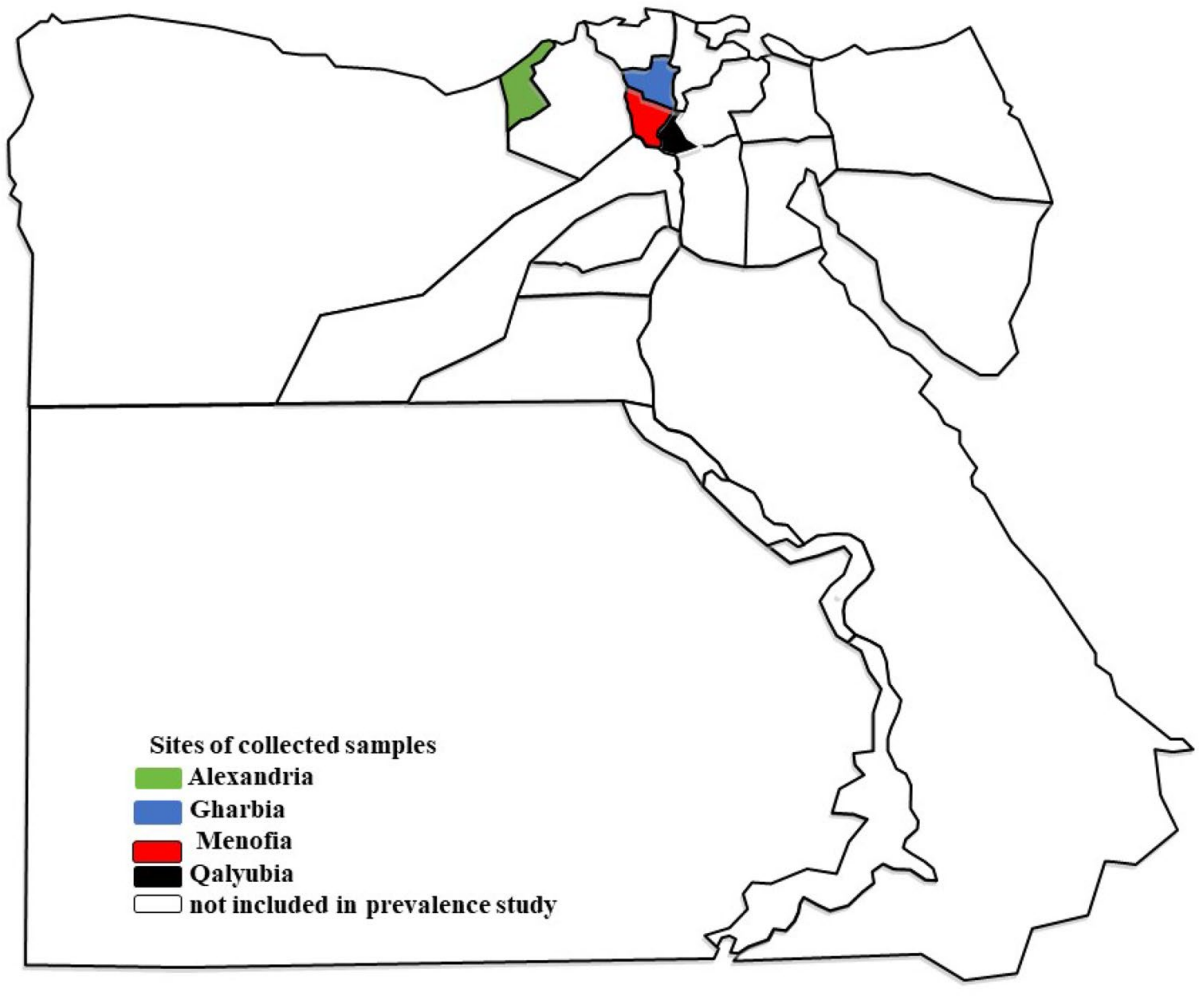
Geographical distribution of governorates under the study. Map was generated using QGIS software ver. 3.18.3. (https://qgis.org/en/site).

The study area includes four north Egyptian governorates as Alexandria, Gharbia, Menofia, and Qalyubia, situated geographically at 31°12′N 29°55′E; 30.867°N 31.028°E; 30.52°N 30.99°E and 30°25 N to 31°13 E, respectively, Fig. 1. The high temperature representing the climate of selected governorates in summer (average 35 °C) and low temperature (average 15 °C) with little rain in winter allowing vast grazing areas.

### Sample collection and preparation

The sample size was calculated using Cochran’s formula^19^ as follow:

$$ n = Z^{2} \frac{{p(1 - p)}}{{e^{2} }} $$

where n is the sample size, *Z* is the statistic corresponding to level of confidence (95% Confidence interval), *p* is expected which was 36.1% according to the previous rate reported by El-Ghayash^20^, *e* is precision that was 5% and 95% confidence interval. The enrolled sheep were classified by governorates based on the estimated number of sheep in each governorate provided from the governorate's Animal Wealth Development Sector. The enrolled sheep were chosen at random from various geographic locations within governorates.

This seroprevalence study was conducted during the period from 2017 to 2018. A quantitative analysis of questionnaire responses from sheep owners and veterinarians was used to determine the risk factors associated with neosporosis. Such factors include locality, age (< 1, 1–2, and > 2), sex, season, pregnancy status, and dog contact.

Blood samples (5 ml) were collected from each examined sheep's jugular vein using a vacuum tube. The sera were separated from clotted blood after centrifugation at 10.000xg for 15 min, then preserved at − 20 °C until used for the serological analysis.

### Serological analysis

Commercial *N. caninum* Ab ELISA kit (IDexx Laboratories, Westbrook, Maine, USA) was used to investigate the antibodies against *N. caninum* in examined sheep according to the manufacturer's instructions.

The optical density (OD) of samples was measured by ELISA reader at 450 nm. The results were expressed as the ratio of sample absorbance to positive control absorbance (S/P), according to the following formula:

S/P = OD sample − OD negative control / OD positive control − OD negative control.

A sample was considered positive if S/P% value equals to or more than 40%.

### Statistical analysis

The data of the serosurvey was analyzed using SPSS (Ver16, USA). The results were considered significant when the *p*-value was < 0.05. Univariant logistic regression was used to determine the association between seroprevalence of neosporosis in sheep and variables of age (< 1, 1–2 and > 2 years old), sex (ram and ewes), season (spring, summer, autumn and winter), pregnancy status and presence of dogs in contact with examined sheep. Variables with a *P* < 0.05 in the univariable analyses were assessed with the multivariable models to determine risk factors, odds ratio (OR) and confidence interval (CI) of each significant variable in Univariable analyses.

## Results

### Seroprevalence of neosporosis in different governorates

The overall seroprevalence rate of *N. caninum* in the examined sheep was 8.6%. The seroprevalence rate of *N. caninum* among sheep was non-significantly different among the surveyed governorates (*P* = 0.2). The highest seroprevalence rate was observed in Menofia (12.4%, 95% CI: 7.02–29.5), followed by Alexandria (9.6%, 95% CI: 5.2–16.5) and Qalyubia (6.1%, 95% CI: 2.7–12.5), but the lowest prevalence rate was reported in Gharbia (5.8%, 95%CI: 2.1–13.8), Table 1.

**Table1:** Prevalence of anti-*N. caninum* antibodies in sheep in different governorates.

Locality	No of examined animals	No of positive animals	%	95%CI	*P* value
Alexandria	125	12	9.6	5.2–16.5	0.2
Gharbia	85	5	5.8	2.1–13.8	
Menofia	105	13	12.4	7.02–29.5	
Qalyubia	115	7	6.1	2.7–12.5	
Total	430	37	8.6	6.2–11.7	

The result is non-significant at *P* > 0.05.

### Risk factors associated with *N. caninum* infection

The serological data analysis (Table 2) revealed that the seroprevalence rate of *N. caninum* increased significantly with age (*P* = 0.03). The highest seropositive rate was observed among sheep > 2 years (12.7%, 95%CI: 8.4–18.7), whereas low rates (5.2%, 95% CI: 1.9–12.4 and 5.8%, 95% CI: 2.8–11.07) were recorded for sheep < 1 and 1–2 years old, respectively.

**Table 2:** Prevalence of anti-*N. caninum* antibodies in sheep in relation different variables.

Parameter	No of examined animals	No of positive	%	95%CI	*P* value
**Age**					
< 1	95	5	5.2	1.9–12.4	0.03
1–2	155	9	5.8	2.8–11.07	
> 2	180	23	12.7	8.4–18.7	
**Sex**					
Ram	125	4	3.2	1.03–8.4	0.01
Ewe	305	33	10.8	7.6–14.9	
**Season**					
Spring	94	6	6.4	2.6–13.9	0.3
Summer	134	15	11.2	6.6–18.08	
Autumn	116	11	9.5	5.06–16.7	
Winter	86	5	5.8	2.2–13.6	
**Pregnancy status**					
Pregnant	245	24	9.8	6.5–14.4	0.3
Non-pregnant	185	13	7	3.9–11.9	
**Presence of dogs in contact with sheep**					
Yes	335	35	10.4	7.5–14.3	0.01
No	95	2	2.1	0.37–8.1	

95% CI, 95% confidence interval.

The result is non-significant at *P* > 0.05.

The result is significant at *P* < 0.05.

The seropositivity was significantly (*P* = 0.01) higher among ewes (10.2%, 95% CI: 7.6–14.9) than rams (3.2%, 95%CI: 1.03–8.4). The infection was significantly (*P* = 0.01) increased in sheep raised in close contact with dogs (10.4%, 95% CI: 7.5–14.3) than in sheep raised without dog contact (2.1%, 95% CI: 0.37–8.1). The season and pregnancy status of the examined sheep showed no significant effect on the prevalence of *N. caninum* infection, Table 2.

### Multivariate logistic regression analysis

Three risk factors have been evaluated by Multivariate logistic regression to assess their effect on the prevalence of *N. caninum* among sheep. The results revealed that elder sheep > 2 years old, are 2.4 times as likely to be infected than sheep < 1-year-old. Ewes are 3.3 times as likely to be infected than rams (95% CI: 1.2–9.3). Sheep raised in contact with dogs are 4.9 times as likely to be infected with *N. caninum* than sheep experienced no dog contact (95% CI: 1.2–20.2) (Table 3).

**Table 3:** Risk factors associated with seroprevalence of *N. caninum* in sheep according to logistic regression analysis.

Risk factor	Comparative parameter	Odds ratio (OR)	95% CI
Age	< 1	ref	
	1–2	1.1	0.36–3.4
	> 2	2.4	0.96–7.1
Sex	Ram	ref	
	Ewe	3.3	1.2–9.3
Dog contact	Yes	4.9	1.2–20.2
No dog contact	No	ref	

95% CI, 95% confidence interval; OR, odds ratio.

## Discussion

Abortifacient pathogens infecting small ruminants in Egypt include *Toxoplasma gondii* ^21^, *Brucella* spp.^22,23^, *Coxiella* *burnetti,* and *Chlamydia psittaci*^24–29^, leading to significant economic losses. Few studies investigated the prevalence of *N. caninum* in sheep worldwide when compared to those of the other animals. Moreover, the information about the epidemiological situation of neosporosis in Egypt is scarce as there are only two studies conducted more than a decade ago^12,20^. Therefore, this study revealed the current seroprevalence rate of *N. caninum* among sheep and evaluated its associated risk factors.

The present study's data confirmed the presence of antibodies against *N. caninum* among the examined sheep in different localities with a seropositive rate of 8.6%. The reported rate was broadly in line with those reported in central China, 7.3% and 8.4%^16,30^. On the contrary, it was lower than those reported in Northwest Spain, 10.1%^31^; the Czech Republic,12%^32^; Egypt, 36.1%^20^ and 25.6%^13^; Italy, 44.4%^9^; São Paulo, Brazil, 59.23%^33^; Minas Gerai State, Brazil, 54%^34^, and Northern Jordan, 63%^35^ and higher than those of the other rates recorded in Turkey, 2.1%^36^; Australia, 2.2%^37^; and Slovakia, 3.7%^38^. The variation in the disease's prevalence may be attributed to different geographical or ecological factors, sheep breeds, rearing systems, or survey timing or technique^8,16,23^.

Interestingly, the seropositive rate of *N. caninum* among sheep in the current study was remarkably increased with the age of animals that come in agreement with previous studies^33,39^. Such age-related observations may be due to the high infection rate (27.6% ) of *N. caninum* among Egyptian stray dogs^20^ playing a substantial role in the horizontal transmission of infection between animals^16^ or simply because of the increased probability of exposure to the sporulated oocysts of *N. caninum* by time.

The analysis of the sex factor in this study revealed that the seropositive rate increased significantly in ewes rather than in rams. A similar finding was reported by Wang, et al.^16^, which is possibly related to different hormone levels between rams and ewes^40^ and the stress factors related to pregnancy and lactation. On the other hand, some reports showed no significant difference between rams and ewes and emphasize the role of sex difference^41,42^.

Dogs play an essential role in *N. caninum* transmission where they shed sporulated oocyst into the environment that contaminates the food and water of sheep^43,44^. In the present study, a strong relationship was observed between the number of seropositive sheep and their close contact with dogs. Such findings that come along with previous studies^16,35,43^ confirmed the close association with dogs is a significant risk factor for the occurrence of *N. caninum* infection in sheep.

The present findings demonstrated that the season and pregnancy status of the investigated sheep had no significant effect on the prevalence of neosporosis. Such findings are consistent with the previous result demonstrated that the difference in temperature of day and night weakens the impact of season on the disease's occurrence^30^. Furthermore, *N. caninum* could be transmitted vertically to the fetus and newborn lamb, but the pregnancy has no significant role in the prevalence of neosporosis in sheep^45^.

## Conclusion

The present study updated the situation of ovine neosporosis in four governorates in the North of Egypt after 12 years of missing data and confirms the presence of antibodies (8.6%) against *N. caninum* among sheep flocks. Age, sex, and close contact with dogs have significant roles in the appearance of *N. caninum* infection but season and pregnancy have no important role in the epidemiology of the disease.
